# Trehalose Maintains Vitality of Mouse Epididymal Epithelial Cells and Mediates Gene Transfer

**DOI:** 10.1371/journal.pone.0092483

**Published:** 2014-03-20

**Authors:** Bin Qu, Yihua Gu, Jian Shen, Jinzhou Qin, Jianqiang Bao, Yuan Hu, Wenxian Zeng, Wuzi Dong

**Affiliations:** 1 College of Animal Science and Technology, Northwest A & F University, Yangling, Shaanxi, P. R. China; 2 Shanghai Institute of Planned Parenthood Research, Shanghai, P. R. China; 3 Department of Physiology and Cell Biology, University of Nevada School of Medicine, Reno, Nevada, United States of America; Cincinnati Children's Hospital Medical Center, United States of America

## Abstract

In the present study, trehalose was utilized to improve primary culture of mouse epididymal epithelial cells *in vitro*, and to enhance naked DNA delivery in epididymis *in vivo*. During the six-day culture, the proliferation activity of the cells in the medium with addition of trehalose was higher than that of those cells cultured in absence of trehalose (*p*<0.01). To determine the optimal concentration for cell proliferation, a series of trehalose concentrations (0, 60, 120, 180 mM) were tested, and the result indicated that the cell in the medium with 120 mM trehalose showed the highest proliferation potential. The epididymis epithelial cells were cultured in the medium containing 120 mM trehalose upon 16^th^ passage, and they continued expressing markers of epididymal epithelial cell, such as *rE-RABP*, *AR* and *ER-beta*. Our study also indicated that trehalose concentrations of 120–240 mM, especially 180 mM, could effectively enhance DNA delivery into the mouse epididymis epithelial cell *in vitro*. Moreover, trehalose could induce *in vivo* expression of exogenous DNA in epididymal epithelial cells and help to internalize plasmid into sperm,which did not influence motility of sperm when the mixture of trehalose (180 mM) and DNA was injected into epididymal lumen through efferent tubule. This study suggested that trehalose, as an effective and safer reagent, could be employed potentially to maintain vitality of mouse epididymal epthetial cells during long-term culture *in vitro* and to mediate *in vitro* and *in vivo* gene transfer.

## Introduction

The epididymis is a vital organ of male mammals for natural reproduction, whose epithelium provides a microenvironment facilitating sperm storage and maturation during the transit of sperm. A variety of *in vivo* and *in vitro* models have been recruited in functional studies of epididymal epithelium [1]. As an *in vitro* model, primary cell culture was utilized for epididymal function studies, such as investigations on the interaction between epididymal epithelial cells and sperm *in vitro* [2]–[5]. However, primary culture of epididymal epithelial cells generally display limited proliferation potential, and the cells easily lose the functional characteristics during the extended culture [6], [7]. For an *in vivo* approach, many methods of DNA transfer for tissue could be employed, due to the short experiment period and convenience. The transfected expression via epididymal epithelium or tubule lumen might impact the epididymal physiology and programmed maturation of spermatozoa, which could help elucidate functions of epididymis. Naked DNA transfer has been introduced for the function study of testis [8], but low efficiency hampered its extensive application. Several reports have successfully interfered gene expression in epididymis by epididymal injection with lentiviral shRNA [9], or by electroporation to deliver naked siRNA [10]. However, high voltage of electroporation or viral vehicle itself would cause adverse effects on tissues. Due to their irreplaceable advantages, such as high model fidelity, gene transfer safety and operation simplicity, primary cell culture *in vitro* and naked DNA transfer *in vivo* are still attractive to researchers. Hence, efforts have been made to modify the previous protocols, which could extend the lifespan of cells in primary culture [11], [12] and enhance the efficiency of naked DNA transfer into tissues [13].

Trehalose is a naturally occurring disaccharide containing two glucose molecules bound in an α,α-1,1 linkage, its unique chemical property, non-reducing sugar, which stabilizes cell membranes under various stressful conditions such as heat, freezing, osmotic shock, oxidative stress, and dehydration [14]–[17]. Trehalose could maintain three-dimensional structure of biologic molecules under stress to preserve their biologic functions [18]. Trehalose has been employed in tissue preservation [19]. Recent researches showed that trehalose protected cells against forming autophosome [15], and trehalose could be used as an additive in primary cell culture to enhance their viability [16], [20]. Trehalose has a significant beneficial effect on preserving the developmental potential of animal sperm at temperatures above freezing [21] and during freeze–thawing [22]–[24]. More interestingly, trehalose could not only enhance osteogenesis by promoting long-term bioactivity of BMP-2 *in vitro* and *in vivo* [25], but also improve the levels of transgenic expression after intramuscular injection of naked plasmid *in vitro* and *in vivo* [26], [27]. It suggested that treholase have multiple functions in protecting cells, enhancing cytokines activity and promoting transgenic expression.

In this study, we analyzed the effect of the different concentrations trehalose added into the media on *in vitro* culture of mouse epididymal epithelial cells. We also investigated the possibility of transferring gene into sperm and epididymal epithelial cell simultaneously through trehalose *in vitro* and *in vivo*, which would be potentially valuable in research of reproductive biology.

## Materials and Methods

### Animals and Chemicals

Male BALB/c mice purchased from the Fourth Military Medical University (FMMU, China) were maintained under a constant photoperiod of 12 h light and 12 h dark and received food and water *ad libitum*. All animal protocols used in this study were approved by Fourth Military Medical University Animal Care Committee and Northwest A&F University Animal Care Committee. All chemicals were purchased from Sigma (St. Louis, MO, USA) and culture media were from Gibco (Invitrogen, Grand Island,USA), except where otherwise noted.

### Isolation and Culture of Mouse Epididymal Epithelial Cells

The epididymal epithelial cells were isolated according to the methods as previously reported [28],[29]. In brief, forty day old mice were killed by cervical dislocation, and the epididymes were microdissected in sterile conditions, freed from adhering fat and adventitia, and cut into small pieces (2∼3 mm). For tubule isolation, tissue fragments were treated for 30 min at room temperature in the IMDM (Iscove's Modified Dubecco's Medium) supplemented with the antibiotics and 2 mg/ml IV type collagenase (Invitrogen) and 20 U/ml DNase I (Promega, Madison, USA). The small tubules were collected by gravity sediment. The tubules were then digested for 5 min in 0.125% trypsin-EDTA and filtrated to remove bulk tissues through 70 um nylon membrane filter. Finally,the cells were collected by centrifugation at 600 g for 5 min,washed,and suspended in the culture media. For epithelial cells migration and proliferation, serum-free IMDM medium (v/v) supplemented with the following nutrients and growth factors: 50 U/ml penicillin,50 μg/ml streptomycin, 2 mmol/l glutamine,10 μg/ml insulin,10 μg/ml transferring, 1 mM L -carnitine, 1 μg/ml retinoic acid, 10 ng/ml epidermal growth factor, 10 ng/ml cholera toxin, 10 nM hydrocortisone, 100 nM dihydrotestosterone (DHT). The cells were then placed in 6-well plates and incubated in a humidified chamber at 34°Cwith 5% CO_2_. The culture media were changed every 48 hours.

The monolayer epithelial cells reached confluence after 4∼6 days of culture. Cells were digested to amplified by sub-culturing cells at 2×10^5^ in new 6-well plates coated with extracellular matrix (ECM, Cat# E1270, Sigma). The cells cultured in the whole medium were considered first passage.

For studying its effect, a stock solution of trehalose was added into the above serum-free IMDM medium, and the final concentrations of trehalose were 0, 60, 120 and 180 mmol/l (mM), respectively. The cells were digested using 0.25% tripsin-EDTA and suspended with phosphate-buffered saline (PBS).

### Cell Growth and Cell Cycle Analysis

#### Cell growth curve

For growth curve analysis 2×10^5^ cells were plated in 6-well dishes in IMDM with 0 mM, 60 mM, 120 mM or 180 mM trehalose. On d2, d3, d4 d5 and d6 cells were harvested, resuspended in 0.25% trypan blue and live cells were counted on a hemacytometer using light microscopy. The experiments were repeated triplicate.

#### Cell cycle analysis

The cells were digested by 0.25% trypsin-EDTA for resuspending, and the suspended cells were fixed in the presence of 1% paraformaldehyde and washed 3 times in PBS. Pellets were resuspended in PBS with RNase A (500 μg/ml), propidium iodide (PI) (50 μg/ml) and kept 1 h at 4°C in the dark. The cell suspensions were analyzed using a Beckman Coulter flow cytometer apparatus (Beckman Coulter, Brea, USA).

### Indirect Immunocytochemical and Immunohistochemical Staining

For immunocytochemistry staining, the cells were cultured in a one-chamber slide (Nalge Nunc, Rochester, USA) at 34°C, fixed in 4% paraformaldehyde for 15 min. The fixed cells were washed with PBS, and permeabilized with PBS (pH 7.2) containing 0.3% (v/v) Triton X-100 for 10 min at room temperature. The slides were blocked with 10% (w/v) goat serum in PBS for 1 h at 37°C, washed with PBS, then incubated with monoclonal Anti-Cytokeratin Peptide 18 (CK-18, Cat# C1399, Sigma) antibody mouse monoclonal antibody (dilution of 1∶200) for 12 h at 4°C. The secondary antibody employed was fluorescein isothiocyanate (FITC) ligated goat anti-mouse IgG (Sino-American Biotechnology, Luoyang, China). Control cells received the same treatment except that the primary antibody was replaced with PBS. Immunofluorescence-stained samples were examined with an Axiophot microscope equipped for fluorescence detection (Carl Zeiss, New York, USA). Images were captured with a CCD camera using SPOT RT software version 3.1 (Diagnostic Instruments Inc, Sterling Heights, USA) and then edited with Adobe Photoshop 6.0.

For immunohistochemical staining, paraffin sections from mice epididymis were de-waxed, re-hydrated and washed with PBS. Briefly, sections were treated with 0.3% H_2_O_2_ for 10 min and 0.1% trypsin for 10 min, this and each subsequent step were followed with three washes (15 min) in PBS. Then they were blocked in PBS with 10% goat serum for 1 h in room temperature. All treatments were performed in a humid, sealed container. Primary antibody (rabbit anti-GFP antibody, Cat#, ab290, Abcam, Cambridge, USA) was diluted in block solution to 1∶400 and applied to sections for overnight at 4°C and HRP-conjugated goat anti-rabbit IgG (dilution 1∶200) was incubated for 1 h at room temperature. In the following steps we used ABC Immuno Detects kit according to the manufacturer's instruction. As a negative control, serial sections were subjected to the same procedure with normal rabbit serum replacing the primary antibody. The slides were then counterstained with hematoxylin for 5 sec, rinsed in water, dehydrated, cleared in xylene, and mount with neutral balsam. The slides were examined using Olympus BX-52 microscope (Olympus, Tokyo, Japan).

### Total RNA and Reverse Transcription (RT)-PCR

Total RNA was isolated from epididymis or isolated cells using Trizol™ reagent (Invitrogen) according to the supplier instructions. According to manufacturer's instruction, one microgram of total RNA was reverse transcribed using a synthetic oligo(dT) 20 primer and ReverTra Ace reverse transcriptase (ToYoBo, Osaka, JP). After cDNA synthesis, 1/10 of the reverse transcribed material was subjected to 32 cycles of amplification using TaqDNA polymerase (ToYoBo), and GAPDH was used as internal control [28] in RT-PCR assays. PCR amplification was done using the following program: 94°C for 5 min; 30–40 cycles of 95°C for 30 sec, Tm for 30 sec, 72°C for 30 sec; 72°C for 5 min and cooled to 4°C. PCR products were then separated on a 1.2% agarose gel, visualized with ethidium bromide and recorded using a Fluor-S Multi-Imager densitometer (Bio-Rad Laboratories, Mississauga, Canada). The couples of primers and melting temperatures (Tm) were listed in Table 1.

**Table 1:** Sequence of primers used for RT-PCR.

genes	Sequences	Tm (°C)	Amplicon size (bp)
rE-RABP	F: GATTGCCTTTGCCTCCAAGATG	65	433
	R: AGCCGATTGCAATACCTTCACAC		
AR	F: GAGCGTGCGCGAAGCGATCCAGAA	60	524
	R: TGCTGCCTTCGGATATTACCTCCTGCT		
ERβ	F: CTACCTGGAGAACGAGCCCA	65	567
	R: AAGGCACTGACCATCTGGTC		
EGFP	F: GCCGACAAGCAGAAGAACGG	57	157
	R:CGGACTGATGGCTCAGGTAG		
GAPDH	F:AGAGAGAGGCCCTCAGTTGCT	65	77
	R:TGGAATTGTGAGGGAGATGCT		

F, Forward primer; R, reverse primer. GAPDH as an internal standard [28].

### *In Vitro* Transfection of primary Epididymal Cell Cultures

Plasmid pEGFP-C1 (Clontech, Mountain View, USA) was used as exogenous DNA in this study, in which CMV promoter can work in a wide variety of mouse tissues and cells and enhanced green fluorescence protein (EGFP) is a reporter gene for DNA delivery into cell. Different final concentration trehalose (0, 60, 120, 180 and 240 mM), 10 μl of Lipofectamine-2000 transfection reagent (Invitrogen) and 4 μg of the pEGFP-C1 vector were dissolved in 0.5 ml RPMI 1640 medium, respectively. After ten-minute incubation at room temperature, the trehalose and Lipofectamine-2000 transfection reagent were mixed with vector, respectively. After twenty-minute incubation at room temperature, the complex was then used for transfection. The cells were seeded into 6-well plates at a density of 2×10^5^ cells/ml 24 h prior to transfection. The cells were washed with RPMI 1640 medium once before transfection. The cells were cultured 12 h in 1 ml serum-free RPMI 1640 medium containing the transfection complex (DNA and trehalose with different final concentration 0, 60, 120, 180 and 240 mM, respectively) in incubator in 34°C. The medium was replaced with above IMDM containing nutrients and growth factors with 120 mM concentration trehalose. Lipofectamine-2000 was used as control transfecting reagent according to instruction, and then the medium was replaced with above IMDM medium factors without trehalose.

### Analysis of GFP Positive cells by Flow Cytometry

The above cells were harvested by using trypsin (0.25% w/v) when they were cultured for 72 h after transfection, and transferred to 50 ml conical tubes and centrifuge at 400 g for 5 min. The supernatant was discarded and the pellets were resuspended in medium (cell culture medium or PBS with 1% bovine serum albumin), and centrifuged again at 400 g and discarded supernatant. And then the cells were resuspended in a small volume of medium and aspirated up and down through a pipette several times to help disaggregate clumps. Finally, the number of cells was counted and resuspended at an appropriate concentration. The percent of green fluorescent cells was monitored in a 580 nm band-pass filter by flow cytometer with WinMDI software version 2.9 (Beckman Coulter).

### Cell viability analysis

Viability of the transfeced cells were determined by MTT (3-4, 5-Dimethylthiazol-2-yl)-2,5-diphenyltetrazolium bromide) analysis according to the previous report [30]. This first time point was assigned as time zero when the above transfection procedure was done. Cell viability was evaluated by incubating the cells with an MTT solution (0.5 mg/ml in culture medium) for 2.5 h at different time points (0, 24, 48, and 72 h) at 34°C. And then the MTT solution was then removed, and the formazan crystals were solubilized with 1 ml dimethylsulfoxide per well. The colorimetric reaction was measured at 490 nm. Three tests were carried out and each treatment was repeated 3 wells on 6-well plate.

### *In Vivo* Transfection of Mouse Epididymal Cells

To analyze whether trehalose could induce pEGFP-C1 (final concentration 50 ng/μl) into receptor mice epididymal cells *in vivo*, the skin covering the testis and epididymis of 24 male adult mice were cut open under anesthesia, the testis and epididymis were gently squeezed out. The complex of trehalose and the vector (Tre-DNA) or the complex of lipofectamine-2000 and the vector (Lipo-DNA) with 0.1% trypan blue (TB) was injected into epididymis by following two ways, respectively. The DNA (pEGFP-C1 vector) with 0.1% TB was injected by same protocol and PBS with 0.1% TB was also injected as control. In the experiments, 50 ng/μl final concentration of the vector was used.

1. The complexes were injected into the interstitial tissues of 6 mice epididymal caput via a sharpened glass microcapillary pipette (GC100T-10, Harvard apparatus,Kent, UK) until epididymal caput displayed TB, respectively.
2. The complexes were injected into the epididymis efferent ducts of 18 mice via a sharpened glass microcapillary pipette under the stereomicroscope until epididymal caput displayed TB, respectively.

At 3^rd^ day after the complexes were injected, the epididymal tissues were collected and Photographed in fluorescent stereomicroscope. The epididymal caput, corput, and cauda were collected for detecting GFP mRNA by RT-PCR. The epididymal caput slides were analyzed for GFP protein expression by immunohistochemical staining. Sperm were acquired in epididymal cauda for following analysis.

### Sperm Collection and Processing

On the 3^rd^ d after injecting by the above two procedures, the sperm samples were obtained from mouse epididymis cauda using the previously described protocol [31]. Briefly, two epididymidis cauda were cut using a small pair of scissors after blood vesicle and adipose tissue removed, and the tissue were placed gently at the bottom of a 1.5 ml microcentrifuge tube containing 1 ml human tubal fluid (HTF, Merck Millipore, Billerica, USA) at room temperature, and then incubated about 2 h for sperm moving to the medium.

For detecting exogenous DNA in sperm, the sperm were washed 4 times using PBS with 20 U/ml DNase I (Promega, Madison, USA) to remove the plasmids attached onto the sperm membrane.

The sperm were prepared for membrane fluidity assay using a slight modification of the previously described protocol [32]. In brief, stock solution of the probes DPH (1,6-diphenyl-1,3,5-hexatriene, 2 mM in tetrahydrofuran) was stored at 4°C in the dark. For labeling, the 2 μM final concentration of DPH was added to the semen suspension (1×10^6^ cell/ml) after thawing. After incubation for 30 min at 34°C, the sperm were pelleted at 1500 g for 3 min, washed twice and resuspended in PBS. The fluorescence anisotropy was immediately measured in a Hitachi F-4010 spectrofluorometer at 30°C. The excitation and emission wavelengths were 360 and 430 nm, respectively. The polarization value of DPH fluorescence was used as a measure of membrane fluidity.

The results were shown in mean±S.D. SNK (Student-Neuman-Keuls) was applied to compare the significant differences. A value of *p*<0.05 or *p*<0.01 was considered as significant difference.

## Results

### Trehalose Helped Long-term Survival and Functional Maintenance of the Mouse Epididymal Epithelial Cells

**Figure 1:**
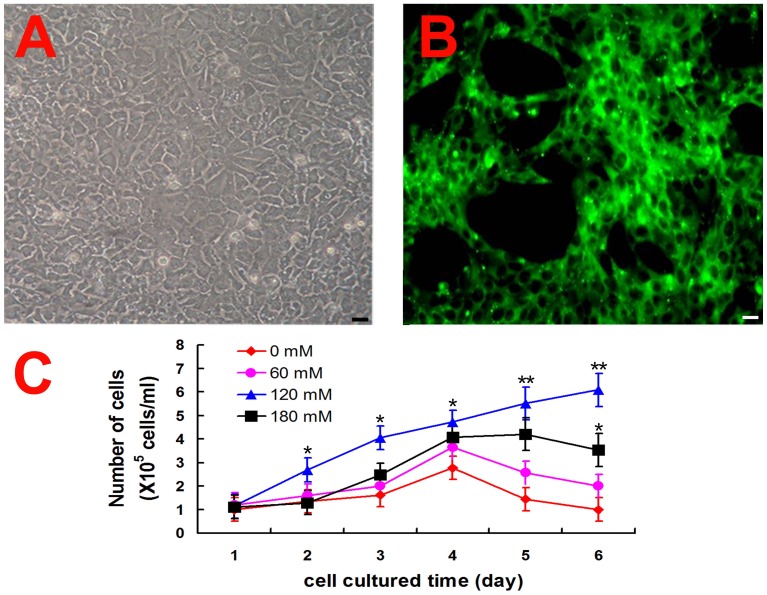
Effects of trehalose at different concentrations on proliferation of mouse epididymal epithelial cells in vitro. A, B) Isolation and CK-18 expression of epithelial cells from forty day mouse epididymis. A: First passage cells cultured in the medium with 120 mM trehalose. B: CK-18 expression of the cells. C) Growth curve of epididymal epithelial cells in the medium with different trehalose concentration. Bars represent means±S.D. * (*p*<0.05) and ** (*p*<0.01) indicated significant difference between 0 mM and other concentrations of trehalose. n = 4.

From 40 day old mice, we isolated epithelial cells from epididymal tubules. Monolayer of cells reached confluency after 3∼4 days of culture in the above serum-free IMDM with trehalose (Fig. 1 A, B). More than 90% of the cells in the monolayer expressed cytokeratin 18 (Fig. 1B), which is a specific marker of epithelial cells. The cells were digested for amplifying by sub-culturing cells at 2×10^5^ in IMDM without FBS in new 6-well plates coated with ECM.

To compare the effects of trehalose concentration on proliferation of the subcultured epididymal epithelial cells, the trehalose with different concentrations (0, 60, 120 and 180 mM) were added to the serum-free IMDM for cell culture,respectively. The cells showed rapid proliferation in the medium with trehalose 120 mM in 6 days (Fig. 1 C). Proliferation activity of the cells decreased in the medium with 60 mM trehalose or no trehalose after cultured for 4 days. On 6th day, the number of the cells in the medium with trehalose 120 mM ((5.9±1.1)×10^5^ cells) was more than that with 180 mM((3.63±0.68)×10^5^ cells) (*p*<0.05) or 60 mM ((2.08±0.74)×10^5^ cells) (*p*<0.05) or without trehalose ((1.06±0.51)×10^5^ cells, *p* = 0.0087<0.01). However, the epididymal epithelial cells almost died during the 2^nd^ passage while they were cultured in the medium without trehalose.

**Figure 2:**
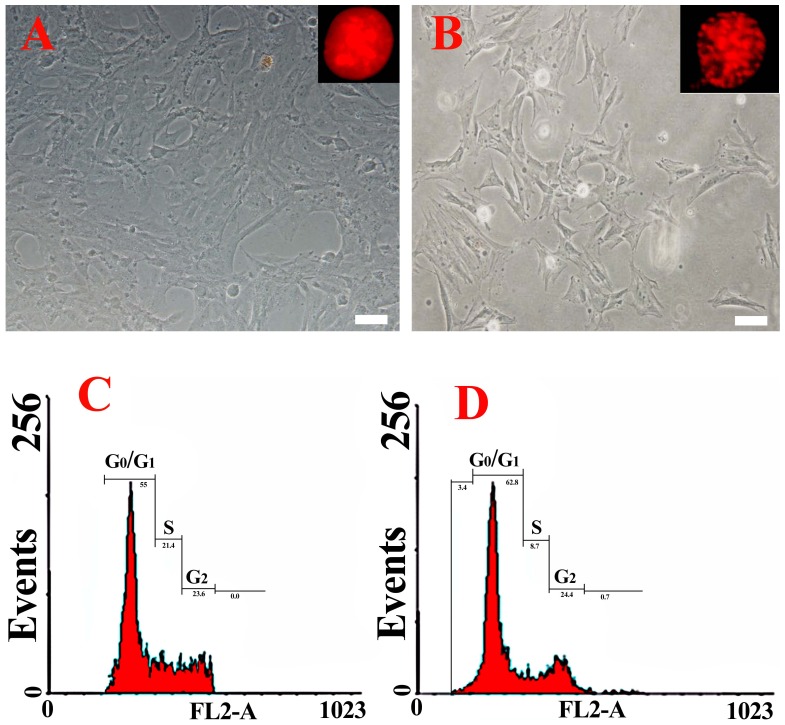
Morphology and cell cycle of the cells at 6^th^ day after subculture in the medium with 120 mM or without trehalose. Cell morphology (A) and cell cycle (C) of the cells in IMDM with 120 mM trehalose; Morphology (B) and cell cycle (D) of the cells in IMDM without trehalose. Bar: 20 μm. Inserts in A, B showed morphology of cellular nuclear using PI staining under fluorescence microscopy (400×).

On 6^th^ day, after the cells were subcultured, the division activities of the cells in medium with 120 mM trehalose were compared with those in medium without trehalose. Under the microscope, the cells demonstrated normal nuclear morphology (Insert in Fig. 2 A) and their side surfaces were tightly linked to those of neighboring cells in the medium with 120 mM trehalose (Fig. 2 A), while the cells ubiquitously had loose nuclear (insert in Fig. 2 B) in the medium without trehalose. The results of flow cytometry analysis indicated that percent of the cells in S-period was the highest (21.4%±0.7%) when cultured in medium with 120 mM trehalose (Fig. 2 C), while the percent of the cells in S-period was the lower (8.7%±0.4%) and there were debris of cells (3.4%±0.2%)when cultured in medium without trehalose (Fig. 2, D). Our study suggests that 120 mM may be the optimal working concentration of trehalose to maintain vitality of the epithelial cells *in vitro*.

**Figure 3:**
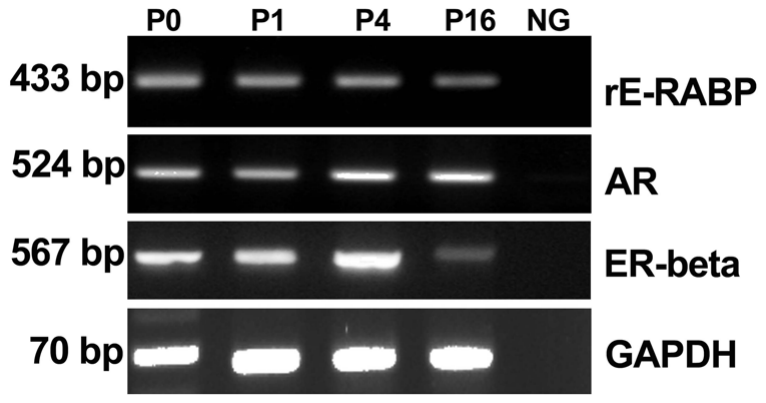
RT-PCR analysis of *rE-RABP, AR, and ER-beta* mRNA levels in isolated epididymal cells (P0), and cells at first, 4^th^, 16^th^ passage culture in presence of trehalose. Cells were cultured in IMDM supplemented with 120(approximately at 6^th^ day of subculture), and then harvested for RNA isolation. mRNA levels of *rE-RABP, AR and ER-beta* were determined by RT-PCR and mRNA of *GAPDH* was used as an inner control. P0, P1, P4, P16 represented the isolated epidiymal cells, the cells at the first passage, the 4^th^ passage, and the 16^th^ passage culture, respectively.

To characterize the epithelial cells cultured with trehalose, some of the special epididymal molecular markers on them were determined, such as *rE-RABP*, *AR*, and *ER-beta*. Our data indicated that these epididymis specific markers were able to be detected in isolated cells (P0), first, 4^th^ and 16^th^ passage cells by RT-PCR, respectively (Fig. 3), which suggested that the isolated epididymal epithelial cells in subculture with trehalose could maintain characteristics of epididymis. To examine the physiologic functions of these cells, co-culture of sperm with the 16^th^ passage cells was performed. We found that the co-cultured sperm could maintain vitality for at least 96 h (data not shown). Our results indicated that the isolated epithelial cells in the present study basically retained both characters and functions of epididymal epithelial cells.

### Trehalose Enhanced Gene Delivery into Mouse Epididymal Epithelial Cells *In Vitro*

**Figure 4:**
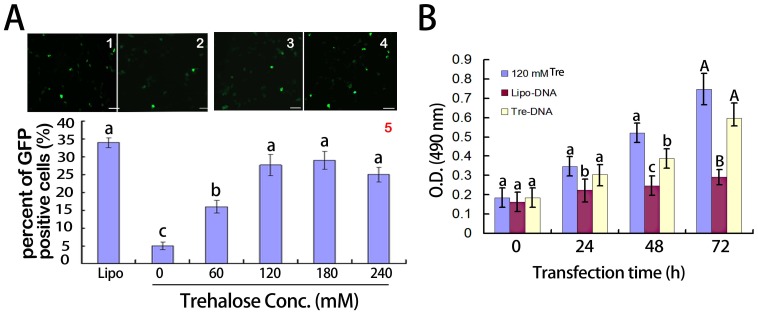
Effects of trehalose on transfer of pEGFP-C1 into the epididymal epithelial cells *in vitro*. A) EGFP positive cells were determined at 72 h after transfection. 1) The green fluorescent cells transfected pEGFP-C1 with lipofectamine 2000; 2) The morphology of cells transfected pEGFP-C1 without lipofectamine 2000 or trehalose; 3) The morphology of cells transfected pEGFP-C1 with 60 mM trehalose; 4) The morphology of cells transfected pEGFP-C1 with 180 mM trehalose; 5) The percent of GFP positive cells was determined by flow cytometry. Lipo represents complex of lipofectamine 2000 and DNA; 0, 60, 120, 180, and 240 represent the complex of different concentration trehalose and DNA, respectively. Different letters indicated significant difference (*p*<0.05), means±S.D, n = 5. B) Effect of 180 mM trehalose-DNA on proliferation of the cells by MTT analysis as described in Materials and Methods. The cells were cultured in the medium with 120 mM trehalose as a control. Lipo-DNA represented the transfection cells with the complex of lipofectamine-2000 and DNA; DNA-Tre indicated that the transfection cells with the complex of 180 mM trehalose and DNA; 120 mM Tre represented control; Different letters (a, b, c) indicated significant differences (p<0.05); and A, B indicated highly significant differences (*p*<0.01). Bars represent means±S.D, n = 5.

**Figure 5:**
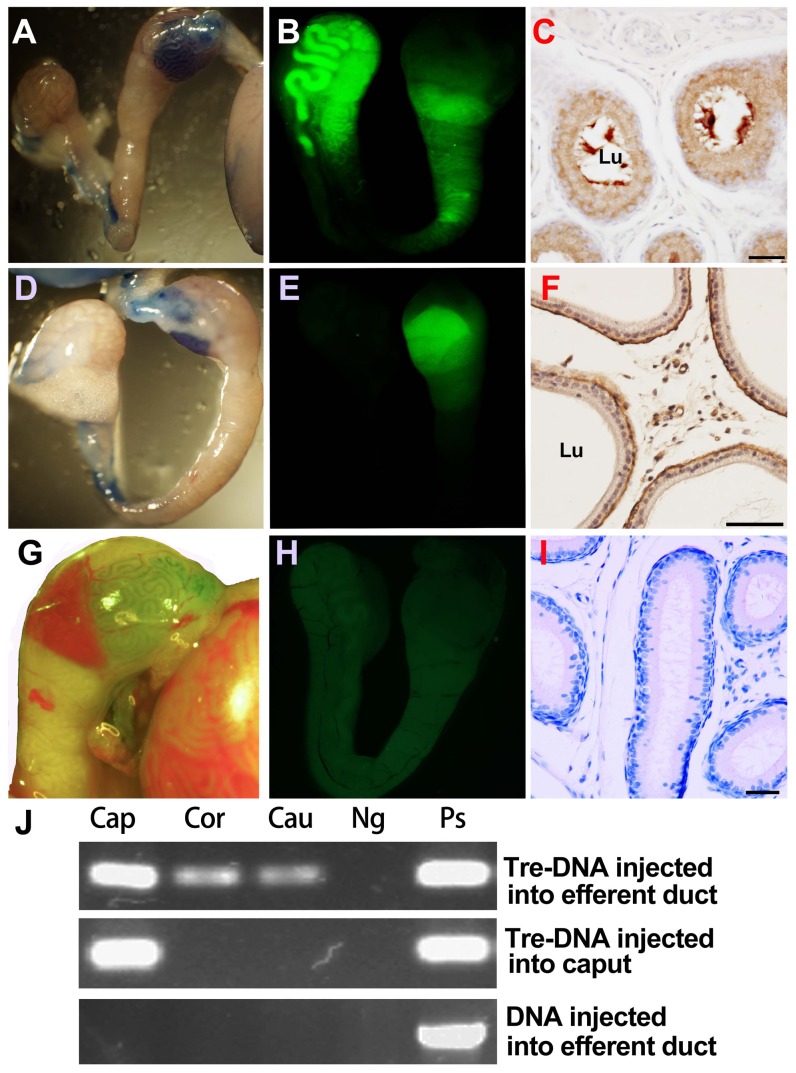
Effects of trehalose on transfer of pEGFP-C1 into the mouse epididymis *in vivo*. A, B, and C) fluorescence appeared in different segments of mouse epididymis at 3^rd^ day after the complex of trehalose-DNA was injected into mouse efferent duct. A) The morphology of mouse epididymis after injecting the complex. B) The mouse epididymis under fluorescence microscopy; C) Localization of GFP protein in epithelial cells and lumen of epididymal caput by immunohistochemistry. D, E, and F) fluorescence only appeared in mouse epididymal caput at 3^rd^ day after Tre-DNA was injected into mouse epididymal caput interstitial tissue. D) The morphology of mouse epididymis after injecting the complex in light view. E) The mouse epididymis under fluorescence microscopy; F) GFP protein expressed in epithelial cells and intercellular cells of epididymal caput by immunohistochemistry. G, H, and I) Little fluorescence appeared in different segments of mouse epididymis at 3^rd^ day after the pEGFP-C1 plasmid was injected into mouse efferent duct. G) The morphology of mouse epididymis after injecting the plasmid in light view. H) The mouse epididymis under fluorescence microscopy; I) No GFP positive signal appeared in the epididymal caput by immunohistochemistry. Bar: 40 μm. J) GFP mRNA expression was detected in mouse epididymal caput, corpus and cauda at 3^rd^ day after treatment by RT-PCR.

By using flow cytometry analysis, we examined the expression of GFP in isolated mouse epididymal epithelial cells, which were previously treated by plasmid p*EGFP-C1* and different concentrations of trehalose (Fig. 4 A). Cells transfected by 120 mM of trehalose showed higher level of GFP expression (27.6%±4.2%) than those treated by 60 mM trehalose (16.1%±1.4%) (*p*<0.05) (Fig. 4 A-5). But the difference was not significant comparing with lipofectamine 2000 (34.7%±3.2%) (*p*>0.05) (Fig. 4 A-5). MTT analysis indicated that the viability of cells treated with the complex of Tre-DNA with 120 mM trehalose was higher than the complex of Lipo-DNA at 72 h after transfection (Fig. 4 B). The results suggested that 120 mM trehalose could not only maintain the cell proliferation for long time but also mediate DNA transfection of mouse epididymal epithelial cells *in vitro*.

### Trehalose Enhanced Exogenous DNA Transfer into Epididymis *In Vivo* and Helped Exogenous DNA Internalization into Sperm

To analyze the expression of exogenous gene in epididymal epithelial cells, the complex of Tre-DNA was injected into the receptor mouse epididymis caput interstitial tissue or efferent duct, respectively. The results showed that exogenous gene *GFP* could be expressed in the epididymis by fluorescence microscopy (Fig. 5 B, E), immunhistochemistry (Fig. 5 C, F) and RT-PCR (Fig. 5 J). GFP expression was detected in epididymis caput, corpus and cauda when Tre-DNA was injected into efferent duct (Fig. 5 A, B, C), while GFP expression could not be detected in corpus and cauda when Tre-DNA was injected into caput interstitial tissue (Fig. 5 D, E, and F) by fluorescence microscopy and immunhistochemistry. The same results were detected when Lipo-DNA was injected into efferent duct or interstitial tissue (Data not shown). However, the fluorescence signals were not detected by fluorescence microscopy and immunhistochemistry when naked plasmid was injected into efferent duct (Fig. 5 G, H, and I) or into caput interstitial tissue (data not shown). No inflammation or necrosis was observed at 3^rd^ day after PBS, trehalose, or the vector was injected into epididymis caput interstitial tissue or efferent duct.

**Figure 6:**
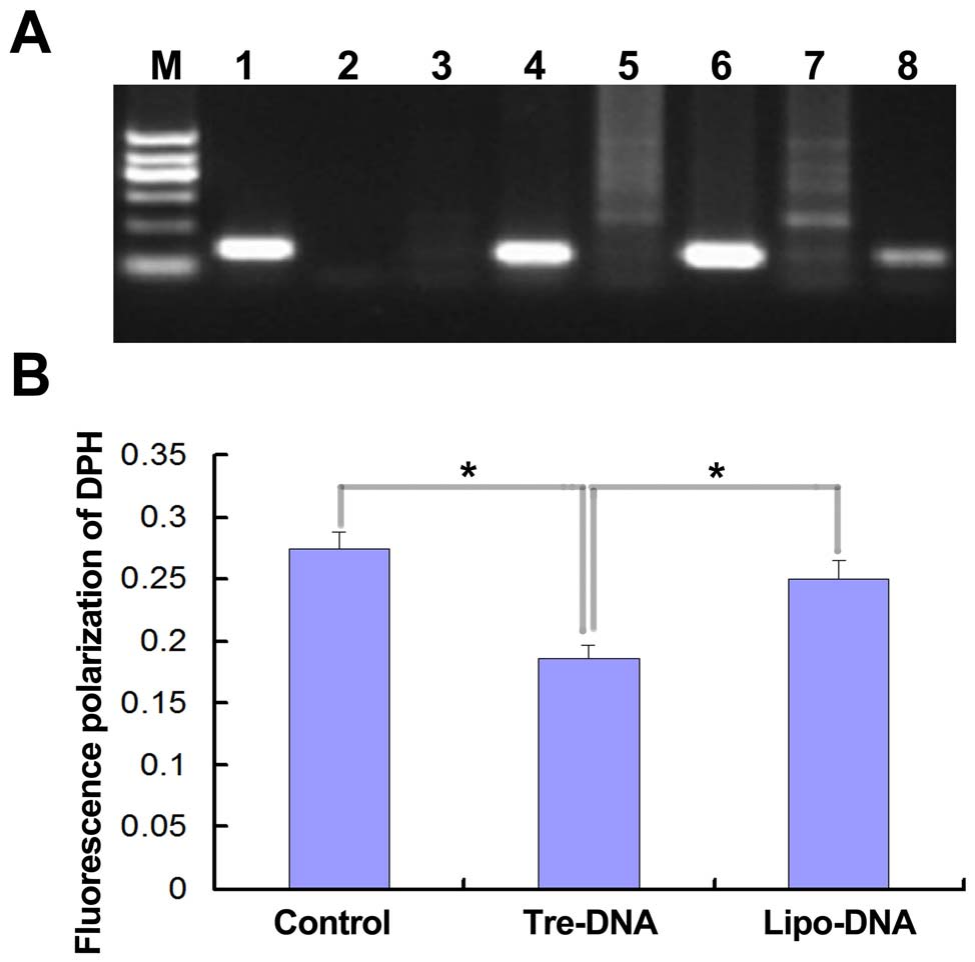
Internalization of plasmid DNA into sperm and analysis of sperm membrane fluidity at 3^rd^ day after the complexes injected into mouse epididymal efferent tubule. A) Detection of internalization of plasmid DNA into sperm by PCR. M: molecular mark; 1^st^ lane: plasmid DNA; 2^nd^ lane: the sperm samples from wildtype mouse; 3^rd^, 5^th^ and 7^th^ lane: the sperm samples from mouse injected Lipo-DNA; 4^th^, 6^th^, and 8^th^ lane: the sperm samples from mouse injected Tre-DNA. B) Membrane futility of sperm was measured by fluorescence polarization of DPH according to Companyo, M et al [32]. The plasma membrane fluidity increasing, while the DPH fluorescence polarization decreasing. Control: sperm from common mouse; Tre-DNA: sperm from mouse injected complex of trehalose and plasmid DNA; Lipo-DNA: sperm from mouse injected complex of lipofectamine 2000 and plasmid DNA. * indicated significant differences (p<0.05,n = 8).

To analyze the effect of Tre-DNA on sperm,epididymis cauda sperm was acquired at 3^rd^ day after injection Tre-DNA into efferent tubule. The DNA of sperm was extracted, and the segment of exogenous DNA can be detected by using PCR with sperm DNA as template (Fig. 6 A). Although it was unknown that how much the plasmid DNA entered into sperm, our result suggested that exogenous DNA could also be transferred into sperm by trehalose.

The membrane fluidity of sperm in original semen was detected by measurement of fluorescence polarization of DPH (Fig. 6 B). We found that the membrane fluidity in sperm increased from Tre-DNA injected mice. The DPH level of sperm from Tre-DNA injected groups was 17.2%±3.7% (n = 3). There was no difference comparing with control treated groups. Whereas it was significantly different comparing with Lipo-DNA injected groups (24.5%±6.7%) (*p*<0.01).

## Discussion

In our study, the presence of trehalose in the medium maintained the viability of the epididymis epithelial cell *in vitro*. Especially, 120 mM trehalose significantly maintained the vitality of the mouse epididymal epithelial cells *in vitro* (Fig. 1 and Fig. 2). The cells with normal nuclear morphology displayed proliferation activity on 6 d in the medium with 120 mM trehalose, while those cells cultured in the medium without trehalose displayed loose nucleus (Fig. 2 insert) that may indicate the formation of apoptotic body. It was consistent with the previous reports that trehalose could protect the cells against apoptosis [15],[17]. The cells expressed key molecular markers such as rE-RABP [33], AR [30] and ER-beta [34] at 1^th^, 4^th^ and 16^th^ passage by RT-PCR (Fig. 3). It indicated the epididymal epithelial cells maintained the common function when they were cultured for long term in medium with trehalose.

Researchers have reported that trehalose enhances transfected expression mediated by naked plasmid *pEGFP-C1 in vitro* [26],[27]. Our results showed that the 180 mM final concentration of trehalose in medium could more significantly improve exogenous EGFP expression in the cells of mouse epididymis than 60 mM trehalose (*p*<0.05) or naked plasmid *pEGFP-C1 in vitro*, though the difference was not significant compared to Lipofectamine-2000 (*p*>0.05) (Fig. 4 A-5). The cells had better viability using complex of 180 mM trehalose and *pEGFP-C1* than complex of Lipofectamine 2000 and *pEGFP-C1* (Fig. 4 B). It was reported that extracellular trehalose, like other small molecules that did not readily cross membranes [35], could be efficiently loaded with membrane-bound pinosomes or endosomes into the cells via fluid-phase endocytosis and pinocytosis [36]. It could be an effective interpretation that trehalose enhanced exogenoue DNA transfer to the epididymal epithelial cells which had the characteristics of endocytosis and pinocytosis [37]. Furthermore, our results showed powerfully that trehalose could protect the epididymal epithelial cells and maintain vitality of the cells against stress of various environmental factors including exogenous DNA. One possible explanation was that trehalose-induced mTOR-independent autophagy enhanced the clearance of autophagy substrates like mutant protein or toxic metabolite [15]. It was entirely plausible that 180 mM trehalose could effectively induce exogenous plasmid transferring into the epithelial cell from epididymis *in vitro*.

Our *in vivo* experiment first demonstrated that exogenous DNA could be delivered into epididymis through injecting Tre-DNA into efferent tubule (Fig. 5), which was an effective measure for study epididymal function. Moreover, we detected that the plasmid was internalized into epithelial cells (Fig. 5 B, E) and spermatozoa (Fig. 6 A) in epididymal cauda on day 3 after Tre-DNA was injected into efferent tubule. The motility of swim-up sperm was not significantly different comparing the mice injected Tre-DNA to the wild-type. The result showed that trehalose could prevent damage of sperm against the exogenous DNA. We also found that membrane fluidity increased in the sperm from mice with injection of Tre-DNA (Fig. 6 B). We acquired the same result when mouse sperm *in vitro* incubated with Tre-DNA (data not shown). It was not clear whether the fluidity change of sperm membrane induced exogenous DNA internalizing into sperm [38]. It was inferred that the plasmids internalized into sperm could be expressed transiently for the specific purpose when the sperm with plasmid were used to fertilize with oocytes, though we did not obtain transgenic offspring when mating was performed between the male mice injected with Tre-DNA and wild type female mice.

In a summary, in the present study, we reported that trehalose was capable to assist absorbance of naked DNA into mouse epididymal epithelial cells and internalization of exogenous DNA into sperm *in vivo*, with a lower cellular toxicity than Lipofectamine 2000. We first established the method of injecting the complex of DNA and transfer reagent into epididymis through efferent tubule of mouse. Further study would identify that trehalose could mediate transgenic expression in mouse epididymis *in vivo* for functional study of epididymal special gene of sperm mature.
